# Multiple trajectories of family adversity and poverty and adolescent self-harm and suicide attempts: findings from the UK Millennium Cohort Study

**DOI:** 10.1186/s13034-026-01023-6

**Published:** 2026-03-14

**Authors:** Yanhua Chen, Michelle Black, Davara Bennett, Ruth McGovern, Helen Sharp, David H. Rehkopf, David C. Taylor-Robinson, Nicholas Kofi Adjei

**Affiliations:** 1 Department of Public Health, Policy and Systems, University of Liverpool, Liverpool, UK; 2 Population Health Sciences Institute, Newcastle University, Newcastle, UK; 3 Department of Primary Care and Mental Health, University of Liverpool, Liverpool, UK; 4 Department of Epidemiology and Population Health, Stanford School of Medicine, Stanford University, Stanford, CA USA; 5 Department of Medicine (Division of Primary Care and Population Health), Stanford School of Medicine, Stanford University, Stanford, CA USA; 6 Department of Pediatrics, Stanford School of Medicine, Stanford, CA USA; 7 Department of Health Policy, Stanford School of Medicine, Stanford University, Stanford, CA USA; 8 Department of Sociology, Stanford University, Stanford, CA USA; 9 Stanford Center for Population Health Sciences, Stanford University, Stanford, CA USA

**Keywords:** Family adversity, Poverty, Self-harm behaviours, Suicide attempts, Trajectory analysis

## Abstract

**Background:**

Exposure to family adversities including domestic violence, parental mental ill-health, and poverty in childhood increases the risk of self-harm and suicide attempts in adolescents. However, few studies have assessed the influence of clustered family adversity and poverty trajectories throughout childhood on self-harm behaviours and suicide attempts.

**Methods:**

In this population-based longitudinal study, we used data on 9316 children from the UK Millennium Cohort Study. Exposure trajectories of poverty and family adversities were characterised using group-based multi-trajectory models (age 9 months−14 years). Multivariable logistic regression models were used to examine the association of trajectories with self-harm and suicide attempts at age 17. Population-attributable fractions were calculated to quantify the contribution of family adversity and poverty to the outcomes at the country level.

**Results:**

Of 9316 participants, 2087 (22.4%) reported self-harm behaviours and 659 (7.1%) had made a suicide attempt. Compared with children experiencing low poverty and adversity, children in the persistent adversity groups were more likely to report both self-harm and suicide attempts; those exposed to persistent poverty and poor parental mental health were particularly at increased risk of self-harm (OR = 1.71, 95% CI: 1.30–2.24) and suicide attempts (OR = 3.98, 95% CI: 2.76–5.74). Overall, we estimated that about 13.2% of self-harm behaviours and 36.9% of suicide attempts were attributable to persistent family adversities and poverty.

**Conclusions:**

Children growing up with persistent exposure to family adversities and poverty are more likely to harm themselves and attempt suicide, particularly those who experience the combination of persistent poverty and long-term poor parental mental health. Early detection of children at risk and intervention such as anti-poverty approaches to prevent long-lasting adversities are key to alleviating risky behaviours in UK adolescents.

**Supplementary Information:**

The online version contains supplementary material available at 10.1186/s13034-026-01023-6.

## Background

Adolescent self-harm and suicide attempts are significant public health concerns associated with the risk of suicide [1–3]. In recent years, rates of adolescent self-harm in the UK have been rising, and a national representative survey of 17-year-olds conducted in 2018–2019 reported a high prevalence of self-harm and suicide attempts: 24% reported self-harm and 7% attempted suicide [4, 5]. Self-harm behaviours, along with suicide attempts, carry significant social and economic costs. Young people who self-harm are at increased risk of later psychosocial difficulties, including mental disorders, substance misuse, and antisocial behaviour, as well as poorer educational, employment, and financial outcomes [6, 7]. Adolescents who attempt suicide often face poorer socioeconomic outcomes in adulthood, including lower earnings and savings, increased reliance on welfare, and a higher likelihood of remaining single [8]. The annual cost of assessing and treating self-harm in general hospitals is estimated at £162 million in England, with psychosocial assessments costing 72% more for under-18s (£392) than for adults (£228) [9]. As self-harm and suicide attempts (i.e., self-harm with suicidal intent) are strong predictors of suicide death [10], identifying risk factors and effective public health and policy interventions are key to implementing effective prevention strategies.

In recognition of the significant public health impact of self-harm and suicide attempts, the Suicide prevention strategy for England (2023–2028) identifies people who have self-harmed as a key priority group and emphasises early intervention to reduce suicide risk [11]. Self-harm and suicide attempts result from a complex interplay of diverse factors across the life course, highlighting the need for a life course approach to better unpick the cumulative risk factors in adolescence [12, 13]. Negative life events and adversity in childhood have received increasing attention in this area. For example, systematic review evidence shows that adolescents with adverse childhood experiences are approximately 2.5 times more likely to have self-injury behaviours and have suicide ideation [14, 15]. Research has also demonstrated that childhood adversity tends to occur in clusters; exposure to accumulated adversity may increase the likelihood of suicide and self-harm compared to experiencing a single adversity [16–18].

The family environment has an important influence on child development and health outcomes. Common family adversities such as domestic violence, parental mental ill-health, and parental substance misuse negatively impact child development and health outcomes, contributing to mental illness, socioemotional and behavioural problems, cognitive disability, drug experimentation, and obesity [19, 20]. According to the UK’s Census 2021, an estimated 751,000 (19.3%) children aged 10 to 15 lived in households where an adult experienced one or more of these factors in the past year [21]. Previous evidence links exposure to such family adversities in childhood with an increased risk of self-harm and suicide attempts during adolescence [22–24]. Recent longitudinal evidence further supports these findings, showing that persistent exposure to parental mental illness is associated with self-harm both in adolescence and early adulthood [25]. In addition, childhood adversities co-occur and cluster over time, with evidence showing that children exposed to multiple and persistent adversities are at higher risk of mortality, hospitalisation and greater use of the health, social care and justice systems [26–28].

However, any policy design on adverse childhood experiences (ACEs) that does not take the socioeconomic context into account is flawed [29]. Family poverty is critical yet often overlooked. In England, one in five children lives in persistent poverty [30]. Poverty may act as an upstream driver of adversity [31, 32]; while family adversities (e.g., parental mental health and substance use) can further contribute to socioeconomic disadvantage, reinforcing children’s exposure to multiple adversities. According to syndemic theory, poverty and other family adversities tend to cluster together, amplifying their cumulative effects [33]. Therefore, building on our previous work on the clustering of family adversity and poverty across childhood [31], this current study aimed to examine the association between trajectories of childhood family adversities and poverty with both self-harm and attempted suicide in adolescents aged 17.

## Methods

### Study design and population

We used data from the UK Millennium Cohort Study (MCS), a nationally representative prospective cohort of children born between September 2000 and January 2002 [34]. The baseline survey, collected at 9 months, tracked children through ages 3, 5, 7, 11, 14, and 17 across seven waves, with participating families at each wave 18,552, 15,590, 15,246, 13,857, 13,287, 11,726 and 10,625. This study used data from all seven waves. Ethical approval for MCS was granted by the National Health Service Research Ethics Committee and informed consent was obtained from parents and children themselves as they grew up; MCS1: South West MREC (MREC/01/6/19); MCS2 and MCS3: London MREC (MREC/03/2/022, 05/MRE02/46); MCS4: Yorkshire MREC (07/MRE03/32); MCS5: Yorkshire and The Humber-Leeds East (11/YH/0203); MCS6: London MREC (13/LO/1786). No additional ethical approval was required for the present secondary analyses.

### Measures

#### Family adversities and poverty trajectories

The main exposures in this study were the longitudinal trajectories of family adversity and poverty that have been defined in our previous work [31], which identified six adversity trajectory groups experienced by UK children from 9 months to age 14. Family adversity included measures of parental mental illness, domestic violence and abuse within family home, and parental alcohol use and poverty was defined as a household equivalised income below 60% of the national median income, according to the Organisation for Economic Co-operation and Development (OECD) household equivalence scale [35] (see Box 1 in supplementary materials). Using a group-based multi-trajectory modelling technique, six trajectory groups were established [31]: (1) the ‘low poverty and adversity’ group includes children with low overall exposure to childhood family adversity; (2) the ‘persistent poverty’ group includes children with a high probability of experiencing poverty throughout childhood; (3) the ‘persistent poor parental mental health’ group is mainly characterised by high rates of poor parental mental health over time; (4) the ‘persistent parental alcohol use’ group include children with a high probability of being exposed to parental alcohol use throughout childhood; (5) the ‘domestic violence and abuse’ group include children with a high probability of having been exposed to domestic violence throughout childhood; and (6) the ‘persistent poverty and poor parental mental health’ group includes children with high exposure to the co-occurrence of persistent poverty and poor parental mental health over time. For more information on the trajectories, see the online supplementary material (Figure S1 in Supplementary material).

#### Self-harm behaviours and suicide attempts

Self-harm was defined in line with the UK National Institute for Health and Care Excellence as “intentional self-poisoning or self-injury, irrespective of the motivation or apparent purpose of the act” [36]. In this study, self-harm refers to any self-injurious behaviour regardless of suicidal intent, whereas suicide attempts were defined as self-harm with explicit suicidal intent.

Participants aged 17 years were asked questions about self-harming behaviours during the previous year. Specifically, adolescents were asked: “During the last year, have you hurt yourself on purpose in any of the following ways? Response options included: 1. Cut or stabbed yourself; 2. Burned yourself; 3. Bruised or pinched yourself; 4. Taken an overdose of tablets; 5. Pulled out your hair; or 6. Hurt yourself in some other way (such as punching/hitting walls/doors/objects, burning/bruising/pinching/pulling your hair, and taking an overdose of tablets).” The response options for each of these items were “yes” and “no”. Respondents were classified as having engaged in self-harm behaviours if they answered “yes” to one or more of the listed behaviours, with these respondents coded as 1 and all others coded as 0 [4, 37].

Adolescents aged 17 years were asked: “Have you ever hurt yourself on purpose in an attempt to end your life?” to assess suicide attempts during the last year. The response options to each of these items included “yes” (coded as 1) and “no” (coded as 0) [38].

#### Covariates

We considered child’s sex, maternal education (degree plus, diploma, A-levels, GCSE A-C, GCSE D-G, or none), and maternal ethnicity (White or Non-white) when the child was aged 9 months as potential confounding factors associated with the exposures and outcomes, guided by a directed acyclic graph (Figure S2).

### Statistical analysis

First, we identified distinct subgroups of children who shared similar underlying trajectories of poverty and family adversities from 9 months to 14 years of age. For this purpose, we used a group-based multi-trajectory modelling (GBTM) approach [39, 40]. We used the TRAJ procedure in Stata [31]. Second, percentages (%) were used to describe the prevalence of self-harm behaviours and suicide attempts by baseline characteristics.

Third, we used logistic regression to assess the associations between trajectory groups with self-harm behaviours and suicide attempts, deriving odds ratios (OR) and 95% confidence intervals (CI). We tested two regression models: an unadjusted model (crude model) to assess the relationship between predicted trajectory groups and each outcome, and an adjusted model that controlled for covariates (adjusted model). Both models included longitudinal weights at wave 7 that account for attrition and response bias. We also examined whether the associations between trajectory groups and outcomes varied by child’s sex by incorporating interaction terms.

To test the robustness of our results, we conducted additional analyses on missingness. First, we used multiple imputation by chained equation (*n* = 25) with results pooled using Rubin’s rules [41] to address missingness in the confounders and outcomes. Second, we repeated our analysis with the imputed data to see if the imputed analyses show similar results.

Finally, we applied population-attributable fraction [42] (PAF) to estimate the proportion of adolescent self-harm behaviours and attempted suicide that could be prevented at country level if exposure to family adversities and poverty during childhood developmental stages were eliminated or reduced to the levels of children who experience low poverty and adversity. The statistical analyses were carried out using Stata (version 17.0).

## Results

### Study population characteristics

**Fig. 1:**
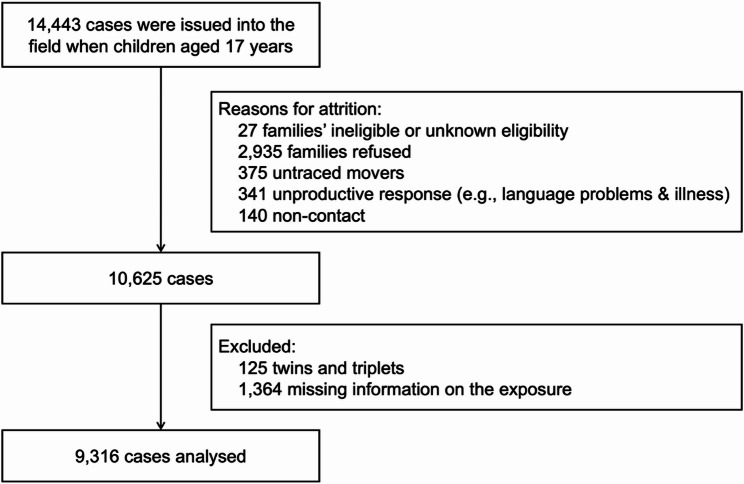
Study flow diagram illustrating the participants included in this study

Of the 10,625 families who were eligible at the age of 17 (wave 7), 9316 cases were analysed after excluding 125 twin and triplet cases and 1364 cases without information on trajectory (Fig. 1). The main models were estimated using a complete-case analysis (i.e., participants with complete observations for exposure trajectory, outcomes and covariates; n = 8613). The prevalence of self-harm behaviours and suicide attempts was 22.4% (2087) and 7.1% (659), respectively (Table 1). More than half of the adolescents (54.9%) had experienced persistent adversity, with persistent poverty the most common form of adversity (21.0%), followed by persistent poor parental mental health (12.0%), persistent parental alcohol use (8.2%), and persistent domestic violence and abuse (3.5%). A combination of both persistent poverty and poor parental mental health impacted 10.1% of children.

**Table 1:** Baseline characteristic and trajectories by self-harm and suicide attempts

	Overall	Self-harm behaviours				Suicide attempts	
	(*n* = 9316)	Yes (*n* = 2087)		No (*n* = 6890)		Yes (*n* = 659)	No (*n* = 8310)
**Childhood family adversities and poverty trajectories**							
Low poverty and adversity	4,204 (45.1)		882 (42.3)		3,180 (46.2)	193 (29.3)	3,864 (46.5)
Persistent alcohol use	763 (8.2)		184 (8.8)		559 (8.1)	36 (5.5)	707 (8.5)
Persistent domestic violence and abuse	330 (3.5)		84 (4.0)		236 (3.4)	30 (4.6)	290 (3.5)
Persistent poor parental mental health	1,122 (12.0)		276 (13.2)		807 (11.7)	105 (15.9)	977 (11.8)
Persistent poverty	1,954 (21.0)		433 (20.8)		1,441 (20.9)	181 (27.5)	1,696 (20.4)
Persistent poverty and poor parental mental health	943 (10.1)		228 (10.9)		667 (9.7)	114 (17.3)	776 (9.3)
**Child’ sex**							
Girls	4,608 (49.5)		1,283 (61.5)		3,153 (45.8)	455 (69.0)	3,973 (47.8)
Boys	4,388 (47.1)		729 (34.9)		3,501 (50.8)	185 (28.1)	4,047 (48.7)
Missing	320 (3.4)		75 (3.6)		236 (3.4)	19 (2.9)	290 (3.5)
**Maternal education**							
Degree plus	1,930 (20.7)		445 (21.3)		1,429 (20.7)	93 (14.1)	1,778 (21.4)
Diploma	870 (9.3)		193 (9.3)		649 (9.4)	59 (9.0)	783 (9.4)
A-levels	931 (10.0)		215 (10.3)		697 (10.1)	61 (9.3)	850 (10.2)
GCSE A-C	2,860 (30.7)		655 (31.4)		2,092 (30.4)	221 (33.5)	2,524 (30.4)
GCSE D-G	813 (8.7)		174 (8.3)		596 (8.7)	80 (12.1)	690 (8.3)
None	1,572 (16.9)		327 (15.7)		1,175 (17.1)	125 (19.0)	1,377 (16.6)
Missing	340 (3.7)		78 (3.7)		252 (3.7)	20 (3.0)	308 (3.7)
**Maternal ethnicity**							
White	7,470 (80.2)		1,773 (85.0)		5,426 (78.8)	568 (86.2)	6,624 (79.7)
Non-white	1,506 (16.2)		233 (11.2)		1,215 (17.6)	71 (10.8)	1,378 (16.6)
Missing	340 (3.7)		81 (3.9)		249 (3.6)	20 (3.0)	308 (3.7)

The missing data on self-harm behaviours is 339 and 347 on suicide attempts

### Associations between exposure trajectories and self-harm and suicide attempts

**Fig. 2:**
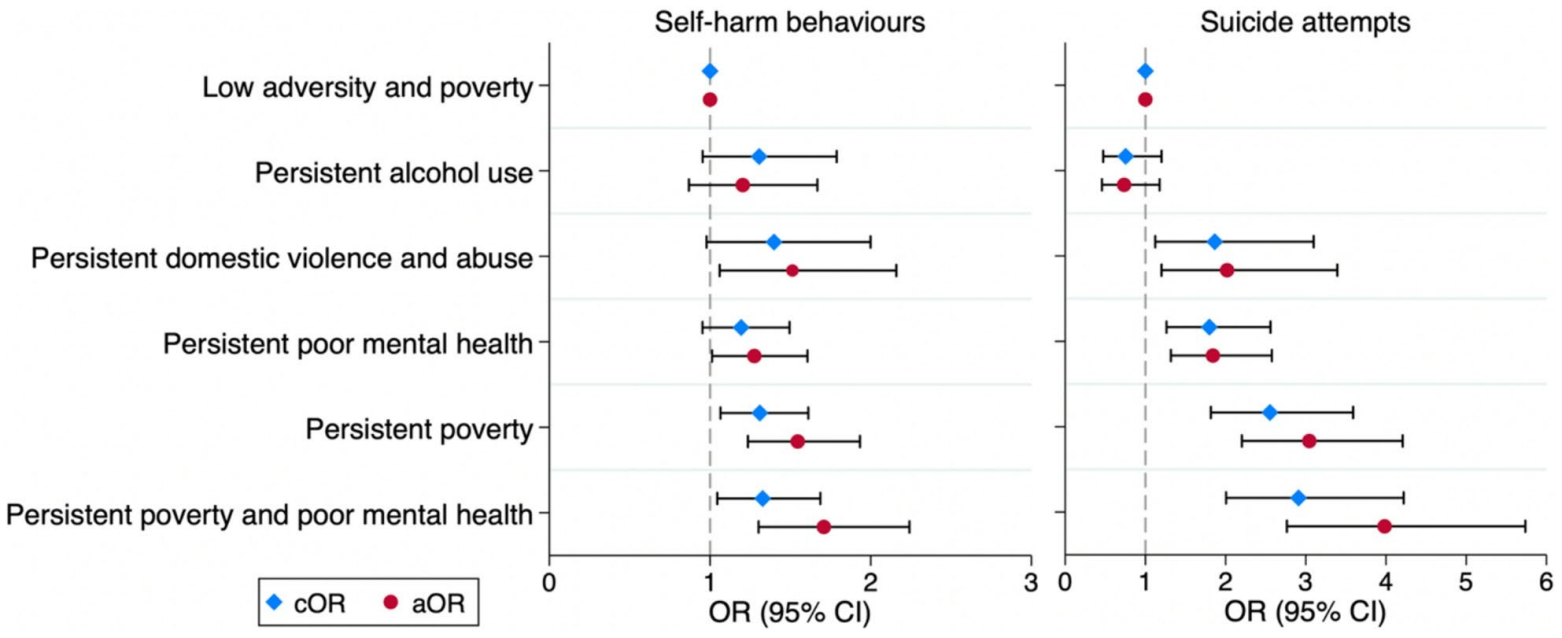
Associations between childhood family adversities and poverty trajectories and self-harm and suicide attempts. cOR for crude model; aOR for model adjusted for child’s sex, maternal education, and ethnicity

The associations of childhood family adversities and poverty trajectories, with self-harm behaviours and suicide attempts, are shown in Table 2; Fig. 2. When compared with children exposed to low poverty and adversity, adolescents had a significantly higher likelihood of engaging in self-harm if exposed to persistent domestic violence and abuse (aOR = 1.51, 95% CI: 1.06–2.16), persistent poor parental mental health (aOR = 1.28, 95% CI: 1.01–1.61), persistent poverty (aOR = 1.55, 95% CI: 1.24–1.93), and the co-occurrence of persistent poverty and poor parental mental health (aOR = 1.71, 95% CI: 1.30–2.24).

Compared with children exposed to low poverty and adversity throughout childhood, adolescents had a significantly higher likelihood of reporting suicide attempts if exposed to persistent domestic violence and abuse (aOR = 2.02, 95% CI: 1.20–3.39), persistent poor parental mental health (aOR = 1.84, 95% CI: 1.32–2.58), persistent poverty (aOR = 3.04, 95% CI: 2.20–4.21) and the co-occurrence of persistent poverty and poor parental mental health (aOR = 3.98, 95% CI: 2.76–5.74). The sensitivity analysis assessing whether the association between the identified trajectory groups and outcomes varied by child’s sex found no evidence of interaction (*p* > 0.05) (Table S1). Following multiple imputation of missing data (Table S2), sensitivity analyses using the imputed datasets produced similar associations to those observed in the main analyses (Tables S3–S4).

**Table 2:** Associations between childhood family adversities and poverty trajectories and self-harm and suicide attempts

Childhood family adversities	Self-harm behaviours		Suicide attempts		
	cOR (95% CI)	aOR (95% CI)	cOR (95% CI)		aOR (95% CI)
Low poverty and adversity	Ref.	Ref.	Ref.		Ref.
Persistent alcohol use	1.31 (0.95, 1.79)	1.20 (0.87, 1.67)	0.75 (0.47, 1.20)		0.73 (0.46, 1.18)
Persistent domestic violence and abuse	1.40 (0.98, 2.00)	**1.51 (1.06**,** 2.16)**	**1.86 (1.12**,** 3.10)**		**2.02 (1.20**,** 3.39)**
Persistent poor parental mental health	1.19 (0.95, 1.49)	**1.28 (1.01**,** 1.61)**	**1.80 (1.26**,** 2.56)**		**1.84 (1.32**,** 2.58)**
Persistent poverty	**1.31 (1.07**,** 1.61)**	**1.55 (1.24**,** 1.93)**	**2.55 (1.82**,** 3.59)**		**3.04 (2.20**,** 4.21)**
Persistent poverty and poor parental mental health	**1.33 (1.04**,** 1.69)**	**1.71 (1.30**,** 2.24)**	**2.91 (2.01**,** 4.22)**		**3.98 (2.76**,** 5.74)**

cOR for crude model; aOR for model adjusted for child’s sex, maternal education and ethnicity. Boldface indicates statistical significance (*p* < 0.05)

### Population attributable fraction (PAF)

**Fig. 3:**
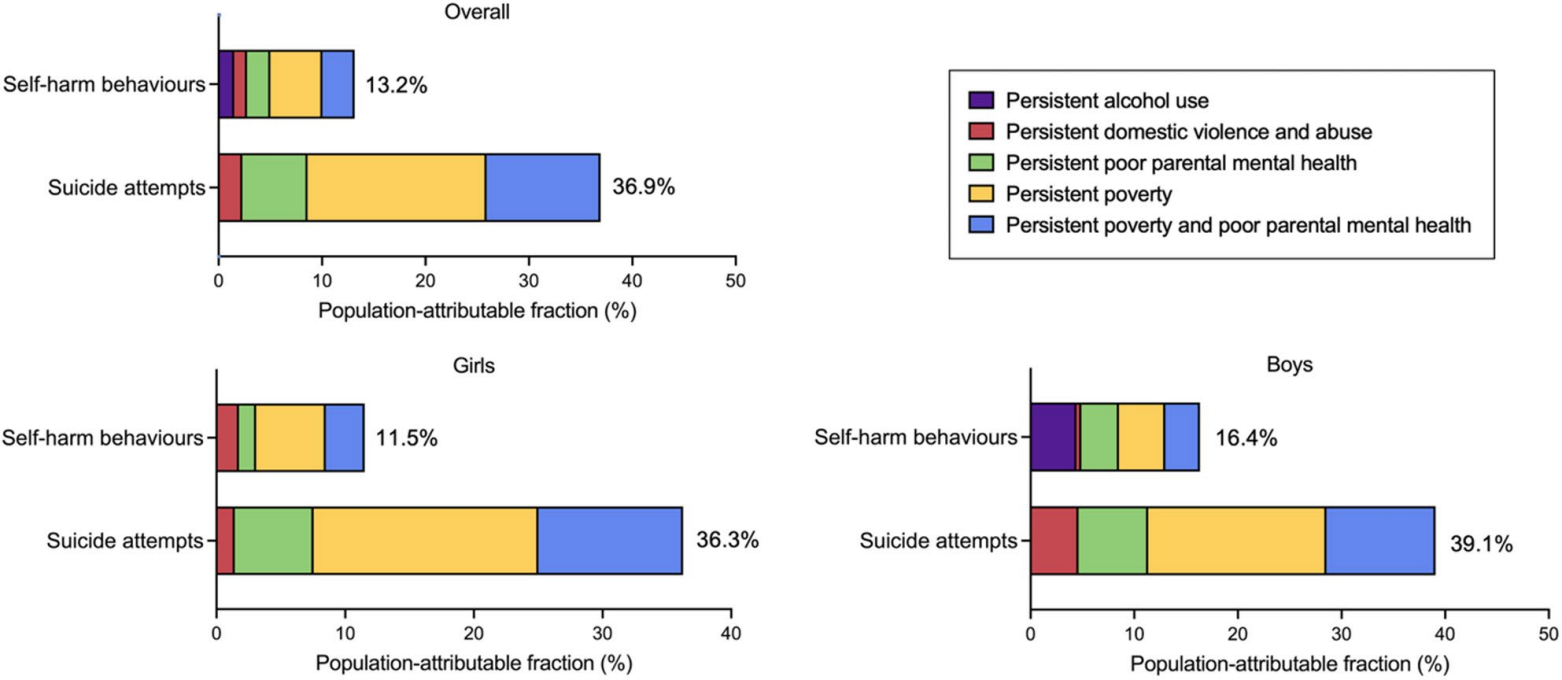
Population attributable fractions of childhood family adversities and poverty on self-harm behaviours and suicide attempts. All model adjusted for child’s sex, maternal education, and ethnicity. Negative contributors to the population attributable fractions are not illustrated in the figures and all detailed contributors (Table S6)

Figure 3 shows the burden of self-harm and suicide attempts attributable to each trajectory group. For self-harm behaviours, the PAF was 1.5% for persistent alcohol use, 1.2% for persistent domestic violence and abuse, 2.3% for persistent poor parental mental health, 5.0% for persistent poverty and 3.1% for persistent poverty and poor parental mental health. For suicide attempts, the PAF was 2.3% for persistent domestic violence and abuse, 6.3% for persistent poor parental mental health, 17.3% for persistent poverty and 11.0% for the co-occurrence of persistent poverty and poor parental mental health. The overall PAFs for self-harm and suicide attempts were 13.2% and 36.9%, respectively.

We further stratified the PAF by sex as a significant gender difference was observed across the trajectories (Table S5). For girls, 11.5% of self-harm and 36.3% of attempted suicide were attributable to persistent childhood poverty and family adversities; for boys, 16.4% of self-harm and 39.1% of attempted suicide were attributable to persistent childhood poverty and family adversities.

## Discussion

Using a large, nationally representative cohort, we examined the associations between long-lasting trajectories of family adversities and poverty on adolescent-reported self-harm and attempted suicide. Persistent adverse family environments, such as domestic violence and abuse, poor parental mental health and poverty - either independently or co-occurring - were associated with an increased likelihood of self-harm and attempted suicide. In particular, adolescents exposed to both persistent parental mental health problems and poverty in childhood were at the highest risk of reporting self-harm behaviours and suicidal attempts.

Previous studies have demonstrated the connection between family environments and self-harm and attempted suicide, showing the impact of differences in adversity types [16, 43] and cumulative effects of disadvantage over time [22, 44] are associated with adolescent self-harm and attempted suicide. Using a novel group-based modelling approach, we identified clusters of children who shared similar trajectories of multiple exposures over time [31], leveraging comprehensive adverse childhood experience data to capture both types and cumulative effects of adversity [45]. Our study extends existing research and is, to our knowledge, the first to examine associations of childhood family adversity and poverty trajectories with self-harm and suicide attempts in adolescence. Furthermore, we calculated PAFs for each trajectory, providing an estimate of their potential contribution to these outcomes and informing the prioritization of public health interventions aimed at preventing self-harm and suicidal behaviours rooted in adverse family environments.

In line with previous findings, the evidence emerging from our longitudinal analysis shows that domestic violence and abuse [46, 47], poor parental mental health [48, 49], and poverty [50, 51] are associated with increased risk of both self-harm and suicide attempts in late adolescence. Noteworthily, no statistically significant associations were observed between persistent parental alcohol use trajectory and self-harm behaviours or suicide attempts. This may be due to other factors, such as parenting style or social support, that affect and modify the pathways linking these associations [52]. In addition, consistent with established evidence on the heightened risk associated with the co-occurrence of adversities [20, 31, 53], our study found that children exposed to a combination of adversities (in this case, persistent poverty and poor parental mental health) had a significantly higher risk of both self-harm and suicide attempts than those exposed to single adversity. The combination of these adversities had the strongest effect, demonstrating the cumulative impact of multiple adverse exposures, highlighting the critical importance of addressing intersecting adversities to mitigate self-harm and suicidal behaviours during adolescence.

It is worth noting that approximately one-third of participants experienced persistent poverty, with about 10% facing additional challenges related to parental poor mental health, which together accounted for over 8% of the PAF for self-harm and more than 28% of attempted suicide. Our analysis supports previous research suggesting that poverty may increase the likelihood of family disharmony and adverse childhood experiences [29, 54], and should be considered a key target for prevention efforts. Evidence from longitudinal and review studies indicates that low household income increases the risk of self-harm and suicide attempts [55, 56]. Moreover, findings from income support and anti-poverty initiatives suggest that improving household finances can enhance parental mental health and children’s emotional well-being [57, 58], which may in turn help reduce the risk of self-harm behaviours. In this current study, persistent poverty combined with poor parental mental health forms a particularly harmful environment for children, elevating the risk of self-harm behaviours and suicide attempts in adolescents. These findings point to the need for a comprehensive, family-centred approach to addressing children’s mental health problems, with a key focus on reducing household poverty and parental mental health problems [59].

This study has several strengths. First, we used secondary data from a large, nationally representative longitudinal cohort in the UK, which collected data on family environment and self-harm and suicidal behaviours from childhood (9 months) to young adulthood (up to 17 years). This allowed us to investigate the influence of childhood family adversity and poverty on self-harm and suicide attempts at a critical transition from childhood to adulthood, providing valuable evidence to inform public health policy in the UK, with potential applicability to other high-income countries. Second, in measuring family environment exposure, we considered various types, cumulative and long-term effects, as well as coexisting patterns, reflecting distinct groups in real-world contexts. This approach explores how adversities emerge, co-occur and persist over time, identifying subpopulations with similar adversity patterns across different developmental stages. In addition, we assessed the robustness of the results by applying multiple imputations and further estimated the impact of family exposure risks using PAF, which help inform the prioritization of prevention strategies to reduce self-harm and suicidal attempts among adolescents. However, a few limitations of this study should be noted. The family exposures (e.g., parental mental ill health, frequent parental alcohol use, domestic violence and abuse) and the outcomes were self-reported information which may be subject to reporting bias. Potential bias may arise due to sample attrition in a large longitudinal study. PAFs assume a causal relationship between exposure and outcome and should therefore be interpreted cautiously. The measurement of self-harm was broad, covering six types of behaviours, but distinguishing between them offers more detailed information. Other potential confounders like biological and genetic factors were not included in our models due to data constraints. For example, we were unable to account for the shared genetic liability between the mental health of parents and that of their offspring, which may partly explain the observed associations. Further research in other samples is encouraged, as the data-dependent nature of the trajectories identified may limit their generalisability.

Our findings are important for policy in the context of rising self-harm and incidence of suicide among adolescents in the UK [60–63]. Addressing the underlying risk factors for these self-harm and suicidal behaviours is imperative for prevention [64]. Our analysis underscores that persistent family adversity and poverty - especially child poverty and parental mental health problems - have an important influence on adolescent self-harm behaviours and suicide attempts. In light of the evidence, policies and interventions should aim to address the mental health needs of both parents and children living in difficult financial situations, with tailored assistance for the most vulnerable families. Family-focused practice is effective, as addressing parental mental health issues effectively is likely to be just as important as providing mental health services directly to the children [59]. Given that these adversities may persist over long periods, sustained support for parents and children from birth onwards appears essential. Identifying, supporting and providing services to those facing adversity should be prioritised [65], with support offered through community-based settings, such as children’s centers, family resource hubs, and schools [66]. Strengthening preventive measures will not only alleviate individual suffering but also reduce the wider public health burden associated with mental health crises amongst young people. Furthermore, policies aimed at income redistribution and poverty reduction [31, 67] may contribute to lowering the rates of youth self-harm and suicide attempts. In order to better inform policy, future research should explore whether reductions in poverty can lead to a decrease in the risk of self-harm, particularly by using causal inference approaches.

## Supplementary Information

Supplementary Material 1

## Data Availability

All data used in this study are publicly available.
