# Supplementary material for: Multiple trajectories of family adversity and poverty and adolescent self-harm and suicide attempts: findings from the UK Millennium Cohort Study
Source: Child Adolesc Psychiatry Ment Health. 2026 Mar 14;20:75. doi: 10.1186/s13034-026-01023-6 (PMC13224490; doi:10.1186/s13034-026-01023-6)
Supplement: Supplementary file 1 — Supplementary Material 1 [file 13034_2026_1023_MOESM1_ESM.docx]

**Supplementary material**

**Box 1.** Description of measurements assessed for trajectory exposures

**Figure S1**. Estimated trajectory groups of family adversity and poverty in the UK Millennium Cohort Study

**Figure S2**. Directed acyclic graph (DAG) of this study

**Table S1**. Associations between childhood family adversities and poverty trajectories and self-harm behaviours, with sex interactions

**Table S2**. Baseline characteristic and trajectories by self-harm and suicide attempts, imputed data

**Table S3**. Associations between childhood family adversities and poverty trajectories and self-harm behaviours, imputed data

**Table S4**. Associations between childhood family adversities and poverty trajectories and suicide attempts, imputed data

**Table S5**. Gender distribution across trajectory groups

**Table S6**. Population attributable fractions of childhood family adversities and poverty trajectories

**Box 1.** Description of measurements assessed for trajectory exposures

- **Parental mental ill health (Child aged 9 months)** – Rutter Malaise Inventory (RMI) scale was used to assess parental mental ill health· A shortened 9-item self-completed version of the RMI measuring depression, anxiety and psychosomatic illness was used· The 9-item short form included items ‘feel tired most of the time’, ‘feel miserable or depressed’, ‘worried about things’, ‘often get into violent rage’ ‘suddenly become scared for no good reason’, ‘easily upset or irritated’, ‘constantly keyed up or jittery’, ‘every little thing gets on nerves and wears you out’, and ‘heart race like mad’· Scores from these items were summed, and we used a validated cut off for mental ill health [‘yes (scores >=4)/no’]·
- **Parental mental ill health (Child aged 3 to 14 years) –** Kessler 6 (K6) scale was used to assess parental mental ill health in the last 30 days asking the responders how often they felt depressed, hopeless, restless or fidgety, worthless, or that everything was an effort· Respondents answered on a five-point scale from 1(all the time) to 5 (none of the time)· We reversed and rescaled all items from 0 to 4 for analysis purposes, so that high scores indicate high levels of psychological distress· We used a validated cutoff widely used in previous studies [‘yes (scores >=6)/no’]
- **Frequent parental alcohol use (Child aged 9 months to 7 years) –** the main responder answered a question about their usual frequency of alcohol consumption (‘*Every day, 5-6 times per week, 3-4 times per week, 1-2 per week, 1-2 per month, less than once a month or never’*)·

Dichotomised: [every day and 5-6 times per week (Yes) vs· 3-4 per week/1-2 per week/ 1-2 per month/never (No)]

- **Frequent parental alcohol use (Child aged 11 to 14 years) –** the main responder answered a question about the usual frequency of alcohol consumption *(‘>=4 times per week, 2-3 times per week, 2-4 times per month, monthly or less, or never’)·*

Dichotomised: [4 or more times a week (Yes) vs· 2-3 per week/2-4 per month/ monthly or less/never (No)]

- **Domestic violence and abuse (Child aged 9 months to 14 years) –** the main responder was asked about the use of physical force by the partner in relationship (‘*Yes, No’*)
- **Poverty (Child aged 9 months to 14 years)** – relative income poverty, defined as household equivalised income of less than 60% of national median household income equivalised according to the Organisation for Economic Co-operation and Development (OECD) household equivalence scale


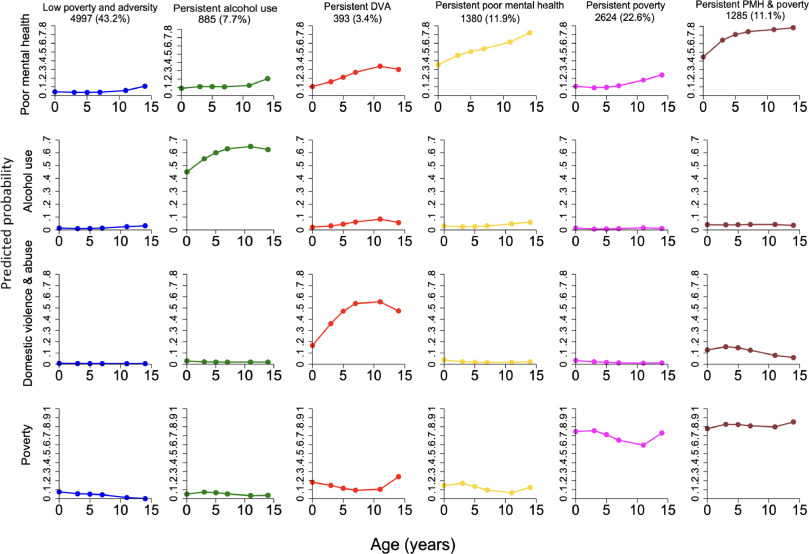


**Figure S1.** Estimated trajectory groups of family adversity and poverty in the UK Millennium Cohort Study

The models were selected based on the Bayesian information criterion (BIC). The BIC values closest to zero denote a better fitting model. The adequacy of the selected model was further judged by: (a) sufficient sample sizes in each identified trajectory group, (b) average posterior probabilities of assignment (AvePP > 0.70), and (c) odds of correct classification based on the posterior probabilities of group membership (OCC > 5.0).

Model Selection Results

| Number of groups | BIC | AIC |
| --- | --- | --- |
| 1 | -85823.59 | -85740.47 |
| 2 | -74515.56 | -74344.13 |
| 3 | -72096.04 | -71836.30 |
| 4 | -69195.50 | -68847.44 |
| 5 | -68265.32 | -67828.95 |
| 6 | -67868.45 | -67343.76 |
| 7 | -67767.82 | -66954.80 |

Note. BIC – Bayesian information criterion; AIC – Akaike information criterion

Model Adequacy Results

| Trajectory group | N | A*ve*PP | OCC |
| --- | --- | --- | --- |
| Low poverty and adversity | 4997 (43.2%) | 0.92 | 16 |
| Persistent alcohol use | 885 (7.7%) | 0.92 | 139 |
| Persistent domestic violence and abuse | 393 (3.4%) | 0.84 | 145 |
| Persistent poor mental health | 1380 (11.9%) | 0.81 | 32 |
| Persistent poverty | 2624 (22.6%) | 0.86 | 21 |
| Persistent poverty and poor parental mental health | 1285 (11.1%) | 0.85 | 48 |

Note. A*ve*PP - average posterior probability; OCC – odds of correct classification.

Membership probability greater than 0.70 and OCC greater than 5 represent a good model fit

**Figure S2.** Directed acyclic graph (DAG) of this study


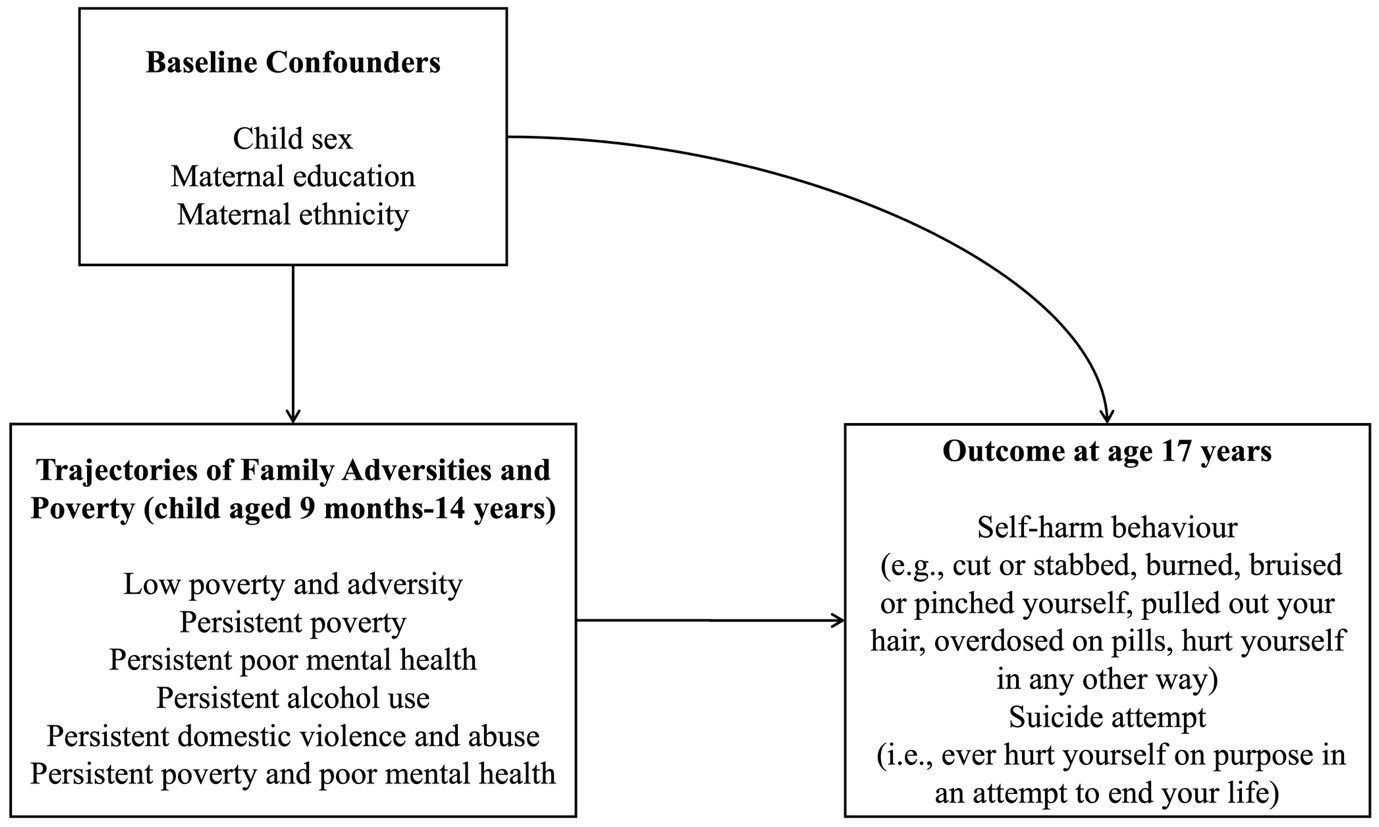


**Table S1.** Associations between childhood family adversities and poverty trajectories and self-harm behaviours, with sex interactions

|  | Self-harm behaviours | Suicide attempts |
| --- | --- | --- |
|  | aOR (95% CI) | aOR (95% CI) |
| Low poverty and adversity | Ref. | Ref. |
| Persistent alcohol use | 1.60 (0.91, 2.80) | 1.00 (0.46, 2.17) |
| Persistent domestic violence and abuse | 1.16 (0.72, 1.88) | **2.86 (1.30, 6.27)** |
| Persistent poor parental mental health | 1.45 (0.98, 2.14) | **1.99 (1.13, 3.51)** |
| Persistent poverty | **1.61 (1.10, 2.34)** | **3.62 (2.09, 6.28)** |
| Persistent poverty and poor parental mental health | **1.65 (1.10, 2.49)** | **3.33 (1.83, 6.06)** |
| Sex (Boys) | Ref. | Ref. |
| Girl | **1.95 (1.58, 2.39)** | **3.03 (1.92, 4.80)** |
| Interaction | Ref. |  |
| Persistent alcohol use#Girls | 0.59 (0.31, 1.12) | 0.64 (0.24, 1.68) |
| Persistent domestic violence and abuse#Girls | 1.62 (0.81, 3.28) | 0.57 (0.21, 1.58) |
| Persistent poor parental mental health#Girls | 0.80 (0.50, 1.28) | 0.90 (0.44, 1.84) |
| Persistent poverty#Girls | 0.94 (0.60, 1.47) | 0.78 (0.40, 1.54) |
| Persistent poverty and poor parental mental health#Girls | 1.09 (0.66, 1.80) | 1.35 (0.64, 2.86) |
| Maternal education (None) | Ref. | Ref. |
| Degree plus | **1.39 (1.04, 1.85)** | 0.95 (0.62, 1.47) |
| Diploma | 1.35 (0.96, 1.89) | 1.71 (0.92, 3.17) |
| A-levels | 1.20 (0.88, 1.65) | 1.02 (0.66, 1.59) |
| GCSE A-C | 1.00 (0.78, 1.29) | 0.97 (0.68, 1.38) |
| GCSE D-G | 1.01 (0.73, 1.40) | 0.95 (0.61, 1.46) |
| Maternal ethnicity (White) | Ref. | Ref. |
| Non-white | **0.54 (0.43, 0.69)** | **0.46 (0.31, 0.68)** |

Note: Boldface indicates statistical significance (p < 0.05). cOR for crude model; aOR for adjusted.

**Table S2.** Baseline characteristic and trajectories by self-harm and suicide attempts, imputed data

|  | **Overall** | **Self-harm behaviours** | | **Suicide attempts** | |
| --- | --- | --- | --- | --- | --- |
|  |  | Yes  (23.3%) | No  (76.7%) | Yes  (7.4%) | No  (92.6%) |
| **Child’ sex** |  |  |  |  |  |
| Girls | 51.2% | 63.9% | 47.4% | 71.2% | 50.4% |
| Boys | 48.8% | 36.1% | 52.6% | 28.8% | 49.6% |
| **Maternal education** |  |  |  |  |  |
| Degree plus | 21.3% | 21.8% | 21.2% | 14.3% | 21.9% |
| Diploma | 9.6% | 9.5% | 9.7% | 9.1% | 9.7% |
| A-levels | 10.3% | 10.5% | 10.3% | 9.4% | 10.4% |
| GCSE A-C | 31.9% | 32.7% | 31.6% | 34.8% | 31.6% |
| GCSE D-G | 9.1% | 8.9% | 9.2% | 12.6% | 8.9% |
| None | 17.8% | 16.8% | 18.1% | 19.8% | 17.6% |
| **Maternal ethnicity** |  |  |  |  |  |
| White | 83.0% | 88.1% | 81.5% | 88.8% | 82.5% |
| Non-white | 17.0% | 11.9% | 18.5% | 17.5% | 11.2% |

Note: Only these three confounders had missingness. Only percentages are presented for multiply imputed data as the ns vary across the 25 imputed dataset.

**Table S3.** Associations between childhood family adversities and poverty trajectories and self-harm behaviours, imputed data

| **Outcomes:** self-harm behaviours | cOR (95% CI) | aOR (95% CI) |
| --- | --- | --- |
| Low poverty and adversity | Ref. | Ref. |
| Persistent alcohol use | 1.30 (0.95, 1.77) | 1.23 (0.90, 1.69) |
| Persistent domestic violence and abuse | 1.39 (0.97, 1.98) | **1.50 (1.05, 2.13)** |
| Persistent poor parental mental health | 1.19 (0.95, 1.49) | **1.29 (1.03, 1.61)** |
| Persistent poverty | **1.33 (1.07, 1.65)** | **1.61 (1.27, 2.04)** |
| Persistent poverty and poor parental mental health | **1.34 (1.05, 1.70)** | **1.75 (1.35, 2.29)** |
| Sex (Boy) |  | Ref. |
| Girls |  | **1.84 (1.57, 2.17)** |
| Maternal education (None) |  | Ref. |
| Degree plus |  | 1.34 (0.99, 1.81) |
| Diploma |  | 1.32 (0.93, 1.87) |
| A-levels |  | 1.18 (0.85, 1.64) |
| GCSE A-C |  | 0.98 (0.75, 1.27) |
| GCSE D-G |  | 0.95 (0.67, 1.35) |
| Maternal ethnicity (White) |  | Ref. |
| Non-white |  | **0.55 (0.42, 0.71)** |

Note: Boldface indicates statistical significance (p < 0.05). cOR for crude model; aOR for adjusted.

**Table S4.** Associations between childhood family adversities and poverty trajectories and suicide attempts, imputed data

| **Outcomes:** suicide attempts | cOR (95% CI) | aOR (95% CI) |
| --- | --- | --- |
| Low poverty and adversity | Ref. | Ref. |
| Persistent alcohol use | 0.76 (0.48, 1.21) | 0.75 (0.47, 1.20) |
| Persistent domestic violence and abuse | **1.86 (1.12, 3.08)** | **2.01 (1.20, 3.36)** |
| Persistent poor parental mental health | **1.78 (1.26, 2.54)** | **1.91 (1.37, 2.65)** |
| Persistent poverty | **2.53 (1.78, 3.60)** | **2.90 (2.07, 4.06)** |
| Persistent poverty and poor parental mental health | **2.89 (2.00, 4.18)** | **3.75 (2.60, 5.40)** |
| Sex (Boy) |  | Ref. |
| Girls |  | **2.77 (2.16, 3.54)** |
| Maternal education (None) |  | Ref. |
| Degree plus |  | 0.93 (0.60, 1.46) |
| Diploma |  | 1.68 (0.90, 3.13) |
| A-levels |  | 1.03 (0.66, 1.63) |
| GCSE A-C |  | 0.96 (0.66, 1.39) |
| GCSE D-G |  | 0.93 (0.60, 1.46) |
| Maternal ethnicity (White) |  | Ref. |
| Non-white |  | **0.45 (0.30, 0.67)** |

Note: Boldface indicates statistical significance (p < 0.05). cOR for crude model; aOR for adjusted.

**Table S5.** Gender distribution across trajectory groups

| **Childhood family adversities** | Girls | Boys |
| --- | --- | --- |
| Low poverty and adversity | 2,076 (50.5%) | 2,033 (49.5%) |
| Persistent alcohol use | 385 (52.3%) | 351 (47.7%) |
| Persistent domestic violence and abuse | 152 (46.6%) | 174 (53.4%) |
| Persistent poor parental mental health | 548 (50.3%) | 541 (49.7%) |
| Persistent poverty | 1,025 (55.2%) | 831 (44.8%) |
| Persistent poverty and poor parental mental health | 422 (48.0%) | 458 (52.1%) |

Note: A Pearson chi-squared test showed a significant difference by sex across trajectory groups (χ² = 19.93, p < 0.01).

**Table S6.** Population attributable fractions of childhood family adversities and poverty trajectories

|  |  | Overall | Girls | Boys |
| --- | --- | --- | --- | --- |
| Self-harm behaviours  (adjusted models*) | Persistent alcohol use | 1.51 (-1.27, 4.22) | -0.47 (-2.52, 1.54) | 4.43 (-1.55, 10.06) |
|  | Persistent domestic violence and abuse | **1.24 (0.08, 2.39)** | **1.75 (0.23, 3.26)** | 0.47 (-1.14, 2.07) |
|  | Persistent poor parental mental health | **2.26 (0.02, 4.45)** | 1.33 (-1.07, 3.67) | 3.64 (-0.64, 7.74) |
|  | Persistent poverty | **5.02 (2.35, 7.61)** | **5.42 (2.04, 8.67)** | **4.46 (0.07, 8.66)** |
|  | Persistent poverty and poor parental mental health | **3.13 (1.43, 4.81)** | **3.02 (1.29, 4.71)** | 3.35 (-0.07, 6.65) |
| Suicide attempts (adjusted models*) | Persistent alcohol use | -1.86 (-4.56, 0.77) | -2.64 (-5.73, 0.35) | 0.05 (-5.37, 5.19) |
|  | Persistent domestic violence and abuse | **2.30 (0.19, 4.36)** | 1.43 (-0.70, 3.53) | 4.59 (-0.26, 9.20) |
|  | Persistent poor parental mental health | **6.30 (2.39, 10.05)** | **6.12 (1.43, 10.59)** | 6.71 (-0.48, 13.38) |
|  | Persistent poverty | **17.31 (11.52, 22.72)** | **17.48 (10.50, 23.91)** | **17.20 (6.94, 26.32)** |
|  | Persistent poverty and poor parental mental health | **11.03 (7.26, 14.64)** | **11.23 (6.94, 15.33)** | **10.53 (3.09, 17.40)** |
| Self-harm behaviours  (crude models) | Persistent alcohol use | 2.17 (-0.53, 4.79) | -0.03 (-2.07, 1.97) | 5.06 (-1.37, 11.09) |
|  | Persistent domestic violence and abuse | 1.00 (-0.15, 2.13) | **1.59 (0.06, 3.09)** | 0.43 (-1.18, 2.02) |
|  | Persistent poor parental mental health | 1.69 (-0.54, 3.86) | 0.59 (-1.84, 2.97) | 3.01 (-1.41, 7.25) |
|  | Persistent poverty | **3.51 (0.70, 6.24)** | 2.36 (-1.07, 5.67) | 2.70 (-1.76, 6.96) |
|  | Persistent poverty and poor parental mental health | **1.88 (0.20, 3.53)** | **1.72 (0.02, 3.40)** | 2.16 (-1.09, 5.31) |
| Suicide attempts  (crude models) | Persistent alcohol use | -1.71 (-4.41, 0.92) | -2.43 (-5.47, 0.53) | 0.02 (-5.38, 5.15) |
|  | Persistent domestic violence and abuse | **2.06 (0.04, 4.03)** | 1.30 (-0.87, 3.42) | 4.55 (-0.29, 9.16) |
|  | Persistent poor parental mental health | **6.35 (2.15, 10.37)** | **5.48 (0.41, 10.30)** | 6.59 (-0.47, 13.17) |
|  | Persistent poverty | **15.96 (9.51, 21.95)** | **15.29 (6.96, 22.87)** | **16.56 (6.70, 25.38)** |
|  | Persistent poverty and poor parental mental health | **9.70 (5.60, 13.61)** | **10.19 (5.64, 14.53)** | **10.24 (2.68, 17.20)** |

Note: * Model adjusted for child’s sex, maternal education, and maternal ethnicity. Boldface indicates statistical significance (p < 0.05).
